# Korea hypertension fact sheet 2020: analysis of nationwide population-based data

**DOI:** 10.1186/s40885-021-00166-2

**Published:** 2021-03-15

**Authors:** Hyeon Chang Kim, So Mi Jemma Cho, Hokyou Lee, Hyeok-Hee Lee, Jongmin Baek, Ji Eun Heo, for the Korean Society of Hypertension (KSH) – Hypertension Epidemiology Research Working Group, Hyeon Chang Kim, Song Vogue Ahn, Sun Ha Jee, Sungha Park, Hae-Young Lee, Min Ho Shin, Sang-Hyun Ihm, Seung Won Lee, Hokyou Lee, Jong Ku Park, Il Suh, Tae-Yong Lee, So Mi Jemma Cho, Hyeok-Hee Lee, Jongmin Baek, Ji Eun Heo

**Affiliations:** 1 Department of Preventive Medicine, Yonsei University College of Medicine, Seoul, 03722 South Korea; 2 Department of Internal Medicine, Yonsei University College of Medicine, Seoul, 03722 South Korea; 3 Department of Public Health, Yonsei University Graduate School, Seoul, 03722 South Korea

**Keywords:** Hypertension, Prevalence, Awareness, Treatment, Control, Antihypertensive medication, Korea

## Abstract

**Background:**

The Korean Society of Hypertension has published the Korea Hypertension Fact Sheet 2020 to provide an overview of the magnitude and management status of hypertension and their recent trends.

**Methods:**

The Fact Sheets were based on the analyses of Korean adults aged 20 years or older of the 2007–2018 Korea National Health and Nutrition Examination Survey (KNHANES) and the 2002–2018 National Health Insurance Big Data (NHI-BD).

**Results:**

Currently, the population average of systolic/diastolic blood pressure was 118/76 mmHg in Korean adults aged 20 years or older showing little change in the recent decade. However, the number of people with hypertension increased steadily, exceeding 12.0 million. Indeed, the number of people diagnosed with hypertension increased from 3.0 million in 2002 to 9.7 million in 2018. During the same period, the number of people using antihypertensive medication increased from 2.5 million to 9.0 million, and the number of people adherent to treatment increased from 0.6 million to 6.5 million. Hypertension awareness, treatment, and control rates increased rapidly until 2007, but showed plateaued thereafter. In 2018, the awareness, treatment, and control rates of hypertension among all adults were 67, 63, and 47%, respectively. However, the awareness and treatment rates were only 17 and 14% among adults aged 20 to 39 years old with hypertension. Among patients treated for hypertension, 61% of them were also using glucose-lowering or lipid-lowering drugs. Among antihypertensive prescriptions, 41% of the patients received monotherapy, 43% received dual therapy, and 16% received triple or more therapy. The most commonly prescribed antihypertensive medication was angiotensin receptor blockers, followed by calcium channel blockers and diuretics.

**Conclusion:**

To achieve further improvement in management of hypertension, we need to encourage awareness and treatment in young adults. It is required to develop tailored prevention and management strategies that are appropriate for and inclusive of various demographics.

**Supplementary Information:**

The online version contains supplementary material available at 10.1186/s40885-021-00166-2.

## Introduction

High blood pressure is the most important modifiable risk factor yet also the biggest contributor to cardiovascular and cerebrovascular diseases [1, 2]. In the twenty-first century, cardiovascular and cerebrovascular diseases mortality accounts for nearly a half of all deaths in the developed regions and for one quarter in the developing regions [3]. The cardiovascular disease mortality rate has been decreasing in Korea; nonetheless, heart disease, cerebrovascular disease, and high blood pressure remain the second, fourth, and tenth in the causes of death [4]. Moreover, due to the rapid aging of the population and improved prognosis from cardiovascular event, the absolute number of patients with cardiovascular disease continues to increase. According to the National Health Insurance Service (NHIS) statistics in Korea, the estimated medical cost of treating hypertension was 3830 billion Korean Won, accounting for 4% of all medical expenses or 16% of medical expenses for chronic diseases [5]. Hitherto, controlling blood pressure is crucial not only to reduce the burden of disease at a societal level but also to improve the quality of life at an individual level. Continuous monitoring of hypertension prevalence and management status should be the first step in reducing its burden. To achieve this, the Korean Society of Hypertension had published its first Hypertension Fact Sheet in 2018 [6] and have been periodically updating it thereafter.

## Materials and methods

The Korea Hypertension Fact Sheet 2020 analyzed two nationally representative datasets. The first one is the Korea National Health and Nutrition Examination Survey (KNHANES) from 1998 to 2018. The KNHANES is a national surveillance system in Korea that assesses the health and nutritional status of noninstitutionalized Korean population since 1998 [7]. The second one is the National Health Insurance (NHI) Big Data from 2002 to 2018. Organized by the National Health Insurance Service (NHIS), the NHI Big Data contains socio-demographics, hospital claims with International Classification of Diseases, 10th Revision (ICD-10) coding, and mortality data of the entire population of Republic of Korea [8]. Previously, the Korea Hypertension Fact Sheet 2018 analyzed adults aged 30 years from the KNHANES data and people of all age in the NHI Big Data. This year, the Korea Hypertension Fact Sheet 2020 included adults aged 20 years or older from both datasets.

The English version of the “Korea Hypertension Fact Sheet 2020” is attached as a supplementary material of this manuscript, and the Korean version is also available at http://www.koreanhypertension.org/reference/guide.

### Analysis of the KNHANES from 1998 to 2018

There have been 7 rounds of KNHANES between 1998 and 2018. The first three KNHANES were conducted every 3 years: KNHANES I (1998), KNHANES II (2001), and KNHANES III (2005). Since 2007, each survey has consisted of 3 years: KNHANES-IV (2007–2009), KNHANES VII (2010–2012), and KNHANES VII (2016–2018). A total of 153,797 adults (average 21,971 per each survey) was analyzed. Hypertension was defined as systolic blood pressure (SBP) ≥140 mmHg, diastolic blood pressure (DBP) ≥90 mmHg, or self-reported use of antihypertensive medication for the purpose of blood pressure control. Awareness rate was defined as the proportion of people with physician diagnosis of hypertension among all people with hypertension. Treatment rate was defined as the proportion of people using antihypertensive drugs for 20 days or more per month among all people with hypertension. Control rate was defined as the proportion of people with SBP < 140 mmHg and DBP < 90 mmHg among 1) all people with hypertension and 2) people treated for hypertension [7, 9]. To evaluate the magnitude and management status of hypertension without the effects of population aging, age-standardized rates were calculated based on the demographics of the Korean population in 2005 according to the Population and Housing Census, Statistics Korea.

### Analysis of the NHI big data from 2002 to 2018

A repeated cross-sectional analysis of 114 million cases of hypertension (assuming 1 case per calendar year if observed more than once) was conducted. While the KNHANES data analysis defined hypertension based on measured blood pressure levels and use of antihypertensive medication, the NHI big data analysis defined hypertension based on diagnosis codes, because the claim database did not have records of blood pressure measurements. Healthcare utilization was defined as at least one health insurance claim for diagnosis of essential hypertension (ICD-10: I10) each year. Treatment of hypertension was defined as at least one health insurance claim for hypertension diagnosis with antihypertensive drug prescription each year. Adherence to treatment was defined as receiving prescriptions of antihypertensive drugs ≥290 days (80%) each year. Antihypertensive drugs were classified into diuretics (DU; thiazide-related diuretics, loop diuretics), beta-blockers (BB), calcium channel blockers (CCB), angiotensin converting enzyme inhibitors (ACEi), angiotensin receptor blockers (ARB), potassium-sparing diuretics (PSD), or others (alpha-blockers, vasodilators, etc.). If the regimen of antihypertensive drug had switched in a year, one with the longest duration was selected as the representative prescription of the patient for the given year. Complication screening rates were calculated for blood test (≥1 serum creatinine test) and ≥ 1 routine urinalysis and/or urine microalbumin test each year, separately. The estimated hypertension burden and management status were additionally calculated for young adults aged 20–39 years.

## Results

### Magnitude of hypertension: based on the KNHANES

The average blood pressure of Korean adults has decreased between 1998 and 2008, but there has been no significant change in the last 10 years. Population mean SBP/DBP level was 118/76 mmHg for Korean adults aged 20 years or older and 119/77 mmHg for adults aged 30 years or older (Supplement, page 9). Over the last 20 years, the age-standardized mean blood pressure levels have decreased yet without significant change in recent years. The age-standardized prevalence of hypertension also modestly decreased from 26.0% (men 29.6%, women 22.3%) in 1998 to 23.5% (men 28.0%, women 18.6%) in 2018 (Supplement, page 10). However, with the rapid aging of the population, the absolute number of people with hypertension has steadily increased; as of 2018, the number has exceeded 12 million. The prevalence of hypertension increased with age in both sex (Supplement, page 11). The sex-specific prevalence was higher in men until the sixth decade and, thereafter, higher in women.

### Hypertension management status: based on the KNHANES

In general, the hypertension management (awareness, treatment, and control rates) has improved significantly over the past two decades. In 2018, among adults aged 20 or older with hypertension, the awareness rate was 67%, the treatment rate was 63%, and the control rate was 47% (Supplement, page 5). However, the degree of management varied greatly by age. All management indices have improved significantly in older adults but not in younger adults. Among patients under age 50 with hypertension, awareness, treatment, and control rates remained below 50%. (Supplement, pages 13–15).

### Healthcare utilization for hypertension: based on the NHI-big data

The number of people diagnosed with hypertension has increased 3.2 times from 3 million in 2002 to 9.7 million in 2018. People receiving antihypertensive prescription has also increased 3.6 times from 2.5 million in 2002 to 9.0 million in 2018. More importantly, the number of people adherent to antihypertensive medication has increased more than tenfold from 0.6 million in 2002 to 6.5 million in 2018 (Supplement, page 18). The use of combination therapy has rapidly increased, with 40.7% using one class, 43.2% using two classes, and 16.1% using three or more classes of antihypertensive drug in 2018 (Supplement, page 20). In 2018, the most commonly prescribed antihypertensive drug class was ARB (71.4%), followed by CCB (60.4%), DU (25.8%), BB (16.0%), ACEi (2.0%), and PSD (1.8%), respectively (Supplement, page 21). ARB (47.3%) and CCB (39.9%) were commonly used for monotherapy, while ACEi/ARB plus CCB was the most frequently prescribed dual therapy regimen (Supplement, page 22).

### Hypertension in young adults: based on the KNHANES and NHI-big data

When extrapolating the KNHANES 2018 data to the entire Korean population, it is estimated that 1.27 million young adults (20–39 years) have hypertension. This accounts for nearly 10% of all adults with hypertension. However, the analysis of NHI-Big Data suggested that, among the 2451 thousand adults with undiagnosed hypertension, 959 thousand (39%) of them were between the age 20 to 39 years (Supplement, page 25). According the KNHANES data from 1998 to 2018, the awareness and treatment rates of hypertension have been steadily rising in older adults but improved little in younger adults. Indeed, the awareness and treatment rates among adults age 20–39 years with hypertension were 17.4 and 13.7%, respectively (Supplement, page 26–27).

## Discussion

The Korea Hypertension Fact Sheet 2020 provides an overview on the magnitude and management status of hypertension in Korea. Although the population average blood pressure and the prevalence of hypertension have changed little over the last decade, the absolute number of people with hypertension have increased steadily-now exceeding 12 million due to population aging. Hypertension management status in Korea had greatly improved until 2007 but remained stagnant thereafter. The greatest novelty of the Korea Hypertension Fact Sheet lies on its generalizability; the KNHANES provides unbiased sampling of the Korean population, and the NHI-Big Data provides medical service uses of the entire nation. However, there are some limitations to be addressed. First, the KNHANES is based on non-institutionalized residents of Korea; thus, it might not include people with severe diseases. Second, the exact collection methods and survey details varied across the KNHANES. Despite standardized protocols and rigorous quality control procedures, such variability may have affected the analysis on secular trends. Third, the NHI-Big Data may not be optimal for identifying disease occurrence and prevalence, because the data have been collected for medical service claims and reimbursement purposes. Fourth, the adherence to antihypertensive medication was evaluated on a prescription basis. Thus, it is possible that adherence was overestimated, because we cannot ascertain whether the drug was actually taken or not. Fifth, we were limited to anonymized datasets; therefore, the data linkage between the two datasets was unachievable.

## Conclusions

Despite the significant improvement in hypertension management over the past few decades, there is still room for further improvement. In particular, the hypertension management status was concerning in younger age groups, regardless of sex. To achieve further improvements in management, it is essential to enable early seeking for disease status and to encourage treatment in young adults. To prevent complications and death from hypertension, emphasizing the importance of treatment adherence and blood pressure control remains a top priority. We also need to develop tailored prevention and management strategies that are appropriate for and inclusive of various demographics.

## Supplementary Information

**Additional file 1.**

## Data Availability

Additional file 1 Korea hypertension fact sheet 2020 – English version. The Korean version is also available at http://www.koreanhypertension.org/reference/guide.
