# Correction to: Korea hypertension fact sheet 2020: analysis of nationwide population-based data

**DOI:** 10.1186/s40885-021-00167-1

**Published:** 2021-03-29

**Authors:** Hyeon Chang Kim, So Mi Jemma Cho, Hokyou Lee, Hyeok-Hee Lee, Jongmin Baek, Ji Eun Heo, for the Korean Society of Hypertension (KSH) – Hypertension Epidemiology Research Working Group

**Affiliations:** 1 Department of Preventive Medicine, Yonsei University College of Medicine, Seoul, 03722 South Korea; 2 Department of Internal Medicine, Yonsei University College of Medicine, Seoul, 03722 South Korea; 3 Department of Public Health, Yonsei University Graduate School, Seoul, 03722 South Korea

**Correction to: Clin Hypertens 27, 8 (2021)**

**https://doi.org/10.1186/s40885-021-00166-2**

In the original publication of this article [1] there were 2 incorrect mentions of KNHANES (Korea National Health and Nutrition Examination Survey). These were as followed:

- **Results**
  - Magnitude of hypertension: based on the **KNHNAES**
  - Hypertension in young adults: based on the **KNHAES** and NHI-big data

These abbreviations have been corrected in the original article.
