# Correction: Korea hypertension fact sheet 2020: analysis of nationwide population-based data

**DOI:** 10.1186/s40885-024-00283-8

**Published:** 2024-10-01

**Authors:** Hyeon Chang Kim, So Mi Jemma Cho, Hokyou Lee, Hyeok-Hee Lee, Jongmin Baek, Ji Eun Heo, for the Korean Society of Hypertension (KSH) – Hypertension Epidemiology Research Working Group

**Affiliations:** 1 Department of Preventive Medicine, Yonsei University College of Medicine, Seoul, 03722 South Korea; 2 Department of Internal Medicine, Yonsei University College of Medicine, Seoul, 03722 South Korea; 3 Department of Public Health, Yonsei University Graduate School, Seoul, 03722 South Korea

**Correction to: Clinical Hypertension (2021) 27:8**

10.1186/s40885-021-00166-2

Following publication of the original article [1], the authors reported an error in the Supplemental Material, page 2, an error in the definition of prehypertension has been noted and corrected as follows:

Original definition

Prehypertension: (1) SBP 130–139 mmHg or (2) DBP 80–89 mmHg; and (3) not meeting criteria for hypertension.

Corrected definition

Prehypertension: (1) SBP 120–139 mmHg or (2) DBP 80–89 mmHg; and (3) not meeting criteria for hypertension.

The original article [1] has been corrected.
