# Supplementary material for: Korea hypertension fact sheet 2020: analysis of nationwide population-based data
Source: Clin Hypertens. 2021 Mar 15;27:8. doi: 10.1186/s40885-021-00166-2 (PMC7958489; doi:10.1186/s40885-021-00166-2)
Supplement: Supplementary file 1 — Additional file 1. [file 40885_2021_166_MOESM1_ESM.pdf]

KOREA HYPERTENSION FACT SHEET 2020

The Korean Society of Hypertension

대한고혈압학회
THE KOREAN SOCIETY OF HYPERTENSION

Korea National Health and Nutrition Examination Survey

Subjects		Adults age 20 years or older / 1998 to 2018
Blood pressure classification	Hypertension	① Systolic blood pressure (SBP) ≥140 mmHg, or<br>② Diastolic blood pressure (DBP) ≥90 mmHg, or<br>③ taking antihypertensive drugs
	Prehypertension	① SBP 120–139 mmHg or<br>② DBP 80–89 mmHg; and<br>③ not meeting criteria for hypertension
Management indices	Awareness rate	Proportion of people with physician diagnosis of hypertension among people with hypertension
	Treatment rate	Proportion of people taking antihypertensives for ≥20 days/month among people with hypertension
	Control rate (among prevalent)	Proportion of people with SBP <140 mmHg and DBP <90 mmHg among people with hypertension
	Control rate (among treated)	Proportion of people with SBP <140 mmHg and DBP <90 mmHg among people taking antihypertensives

Korea National Health Insurance Big Data

Subjects		Adults age 20 years or older / 2002 to 2018
Healthcare utilization	Diagnosis	≥1 health insurance claim for hypertension diagnosis (ICD-10: I10) each year
	Treatment	≥1 health insurance claim for hypertension diagnosis and antihypertensive drug prescription each year
	Adherence	Prescription of antihypertensive drugs ≥290 days (80%) each year
	Blood test	≥1 serum creatinine test each year
	Urine test	≥1 routine urinalysis or urine microalbumin test each year
Treatment for hypertension	Regimen	The combination of antihypertensive classes in a prescription;<br>if the regimen is switched, one with the longest duration is selected for a given year.
	Antihypertensive drug class	Diuretics (DU; thiazide-related diuretics, loop diuretics),<br>beta-blockers (BB), calcium channel blockers (CCB),<br>angiotensin converting enzyme inhibitors (ACEi), angiotensin receptor blockers (ARB),<br>potassium-sparing diuretics (PSD), Others (OTH; alpha-blockers, vasodilators, etc.)

KOREA HYPERTENSION FACT SHEET 2020

Contents

4 Summary of population statistics
6 Summary of treatment statistics

Changes in population mean blood pressure and prevalence of hypertension

9 Changes in population mean blood pressure
10 Changes in hypertension prevalence
11 Changes in hypertension prevalence by sex and age

Changes in hypertension management indices

13 Changes in hypertension awareness rate by sex and age
14 Changes in hypertension treatment rate by sex and age
15 Changes in hypertension control rate (among prevalent) by sex and age
16 Changes in hypertension control rate (among treated) by sex and age

Healthcare utilization for hypertension

18 Changes in the number of healthcare users
19 Changes in co-treatment for dyslipidemia and diabetes
20 Changes in prescriptions of antihypertensive drugs
22 Changes in antihypertensive treatment regimen

Management of hypertension in young adults

25 Summary of hypertension statistics in young adults
26 Changes in awareness rate in young adults
27 Changes in treatment rate in young adults
28 Changes in adherence rate (among diagnosed) in young adults
29 Changes in adherence rate (among treated) in young adults
30 Current management status in young adults

Summary of population statistics (age 20+)

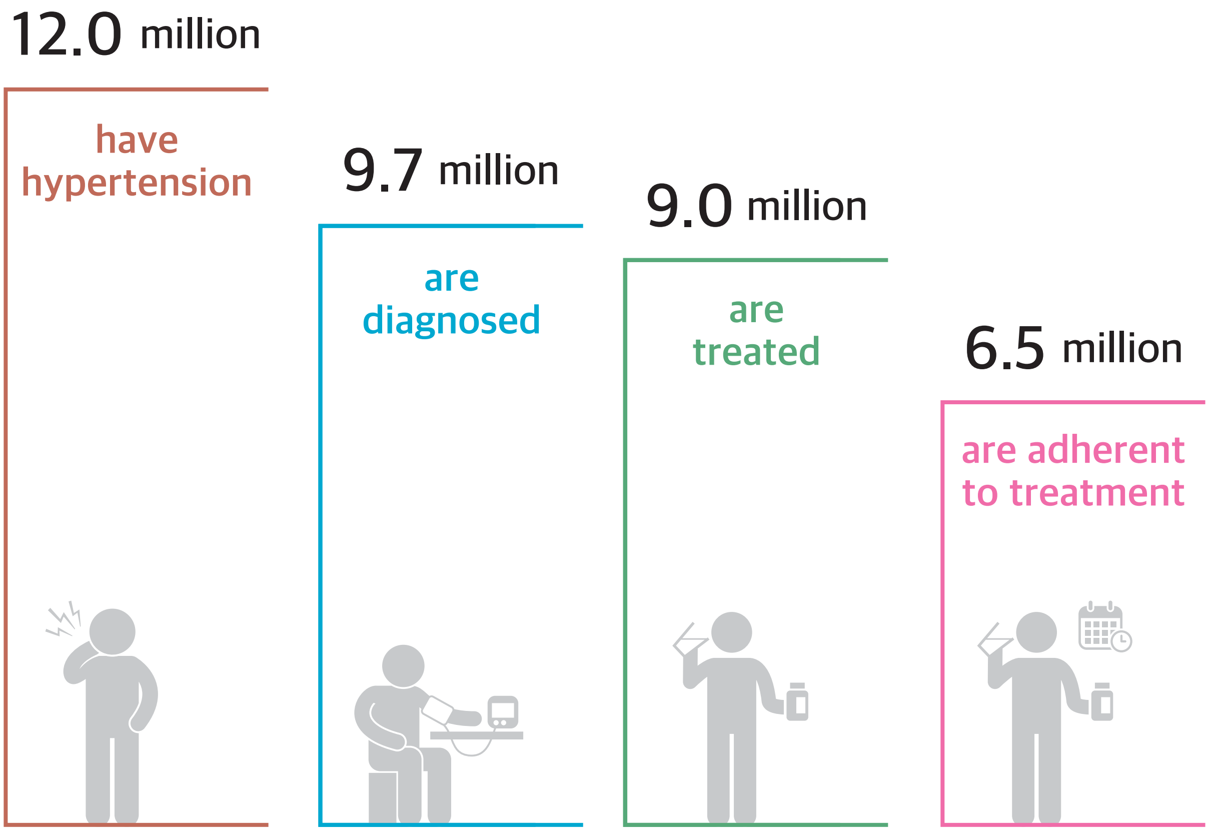

Data source: Korea National Health and Nutrition Examination Survey 2018, Korea National Health Insurance Big Data 2018

Summary of population statistics (age 20+)

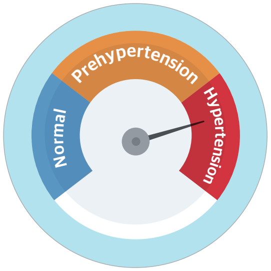

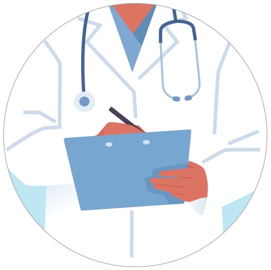

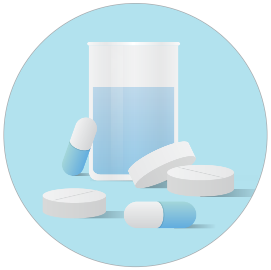

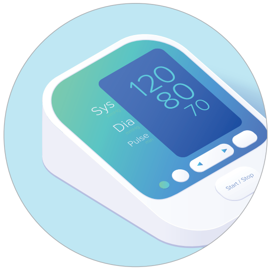

29% have hypertension

67% are aware

63% are treated

47% are controlled

among adults age 20+

among adults age 20+ with hypertension

Data source: Korea National Health and Nutrition Examination Survey 2018

Summary of treatment statistics (age 20+)

Among 9.0 million adults treated for hypertension, %

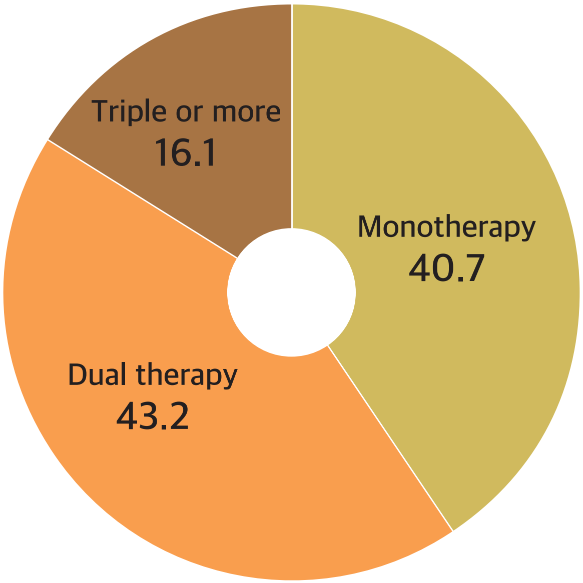

Among 3.7 million adults on monotherapy, %

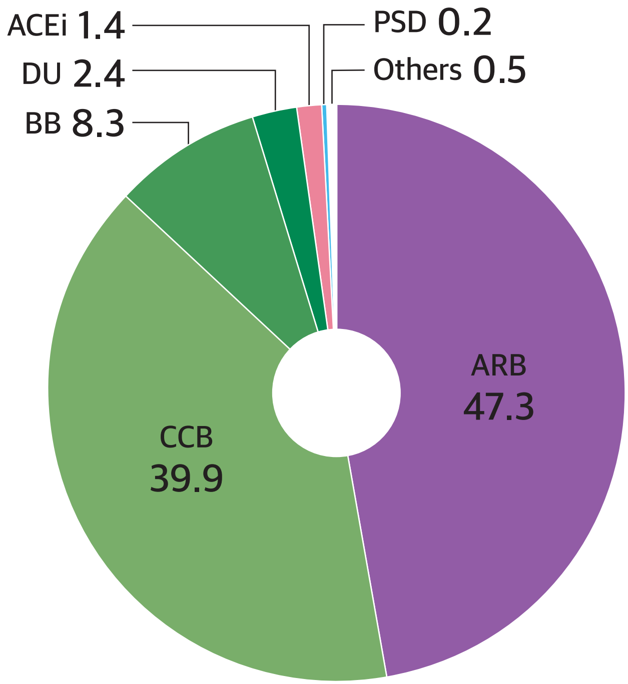

Data source: Korea National Health Insurance Big Data 2018

Summary of treatment statistics (age 20+)

Among 3.9 million adults on dual therapy, %

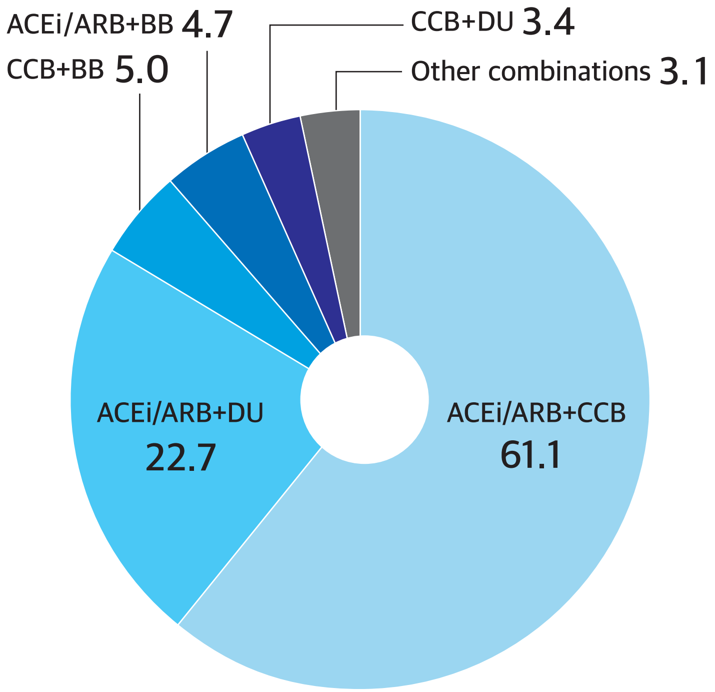

Co-treatment for dyslipidemia and diabetes among 9.0 million adults treated for hypertension, %

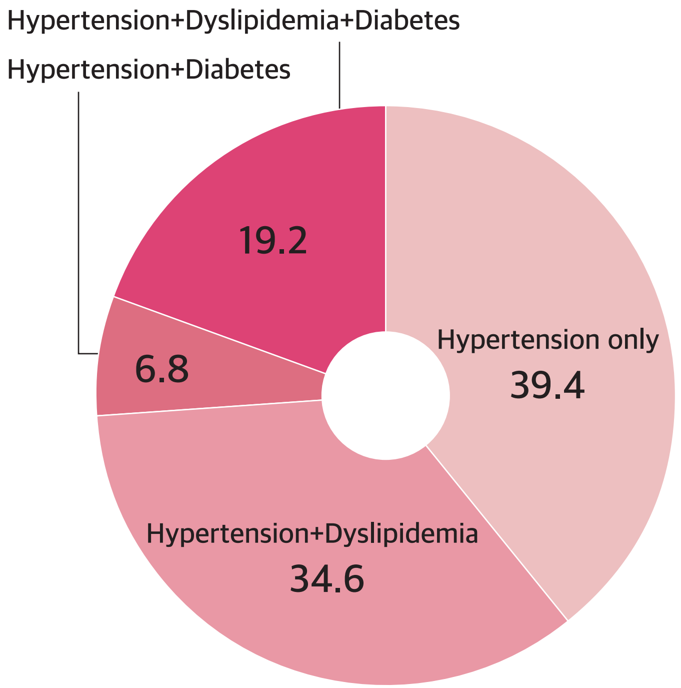

Data source: Korea National Health Insurance Big Data 2018

Changes in population mean blood pressure and prevalence of hypertension

Changes in population mean blood pressure

Changes in hypertension prevalence

Changes in hypertension prevalence by sex and age

Changes in population mean blood pressure

118/76 mmHg (age 20+)

116/76 mmHg (age 20+, age-standardized)
119/77 mmHg (age 30+)
117/77 mmHg (age 30+, age-standardized)

Year 2018

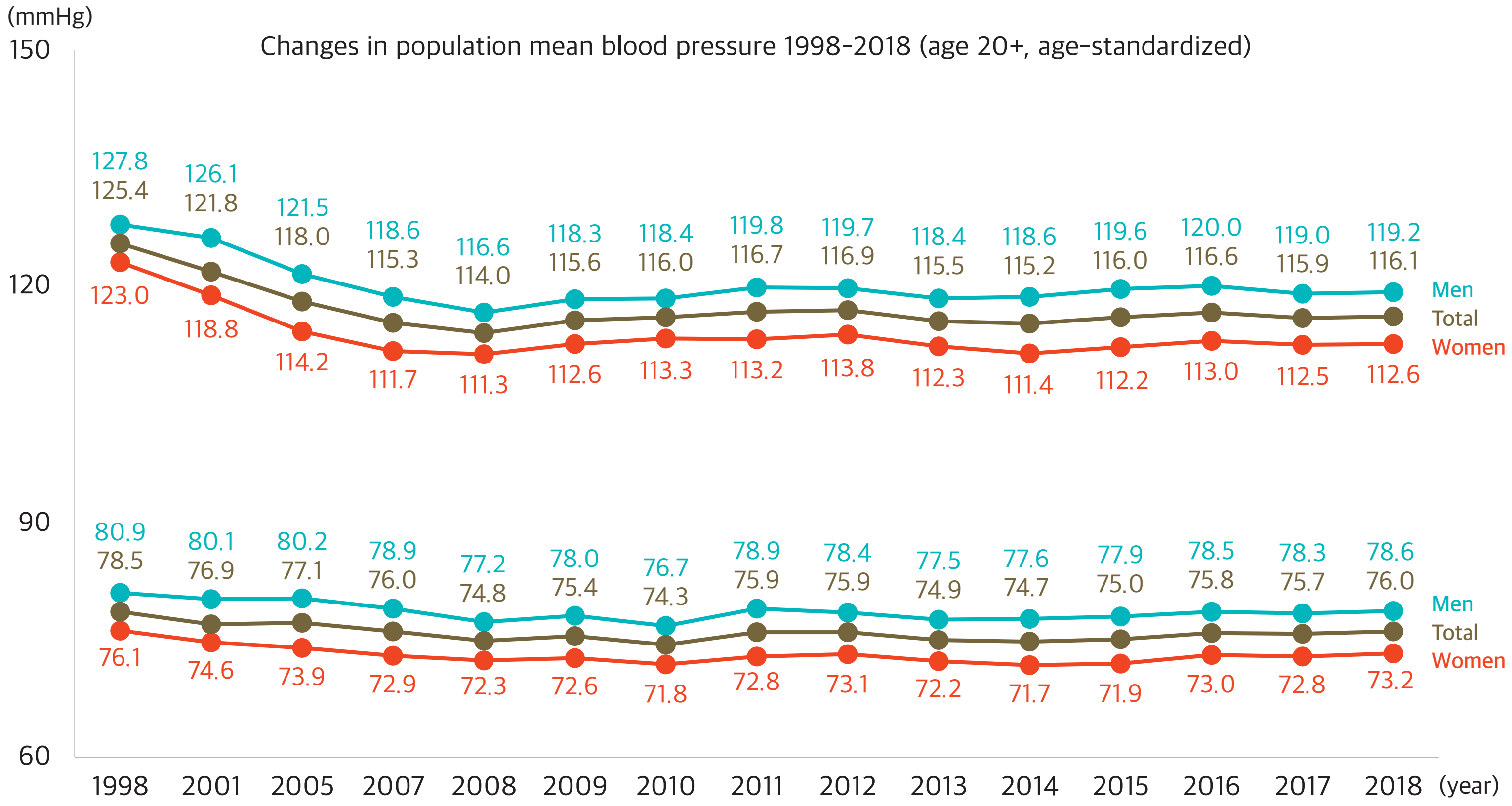

Data source: Korea National Health and Nutrition Examination Survey 1998-2018

Changes in hypertension prevalence

29% (age 20+)

24% (age 20+, age-standardized)
33% (age 30+)
28% (age 30+, age-standardized)

Year 2018

Changes in hypertension prevalence 1998-2018 (age 20+, age-standardized)

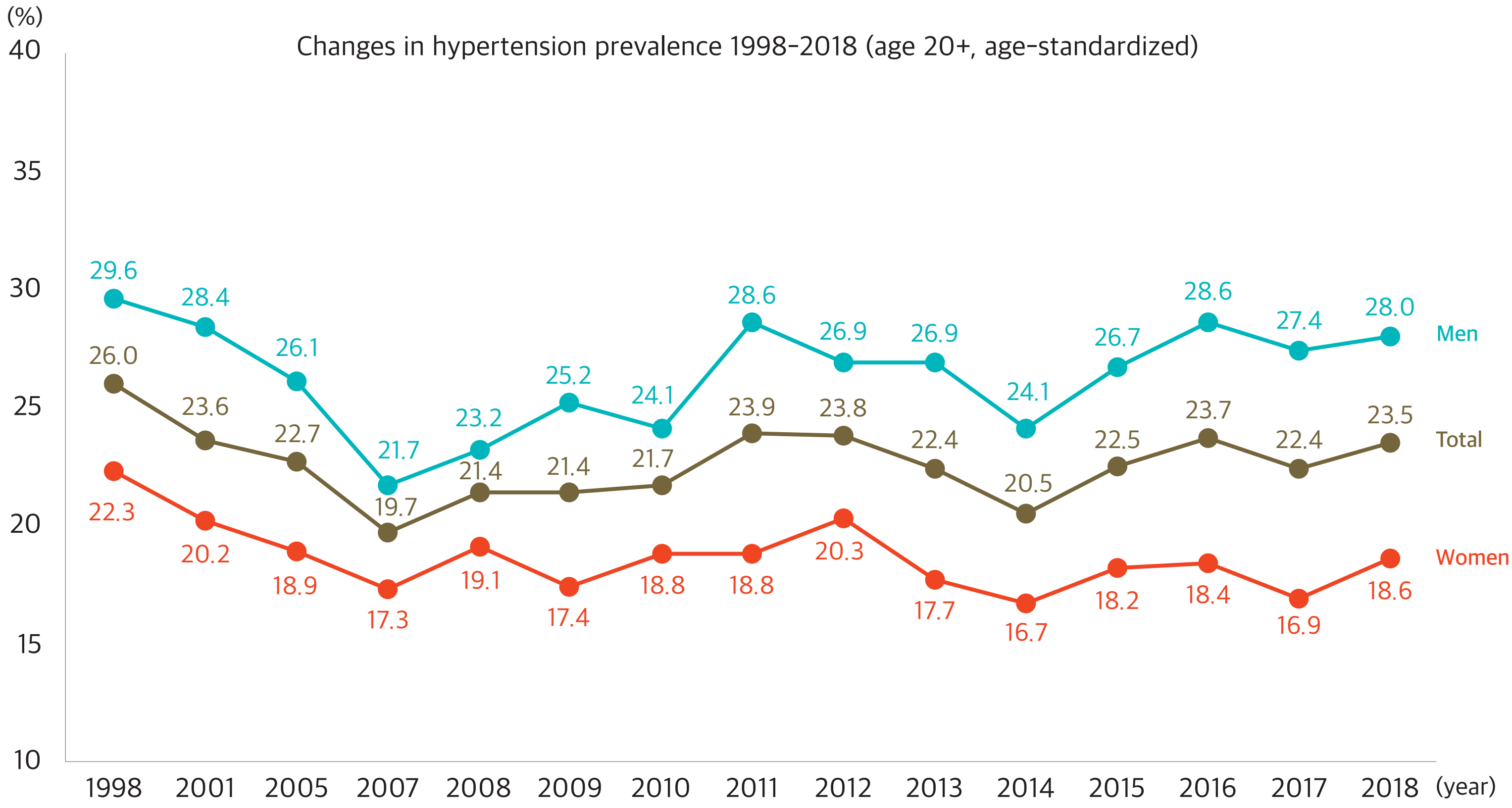

Data source: Korea National Health and Nutrition Examination Survey 1998-2018

Changes in hypertension prevalence by sex and age

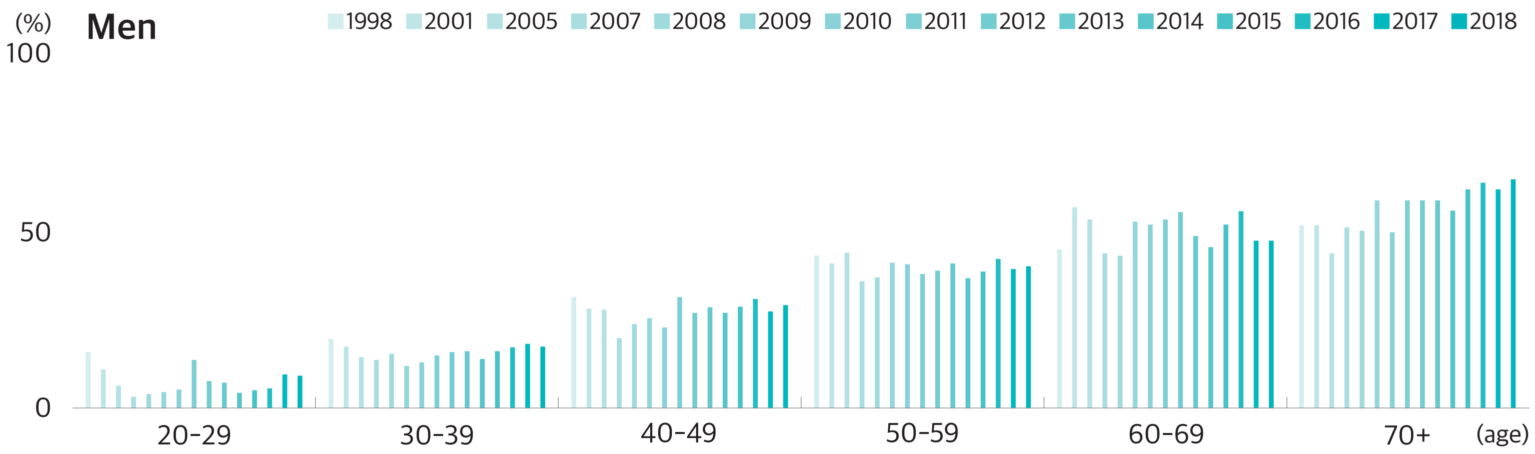

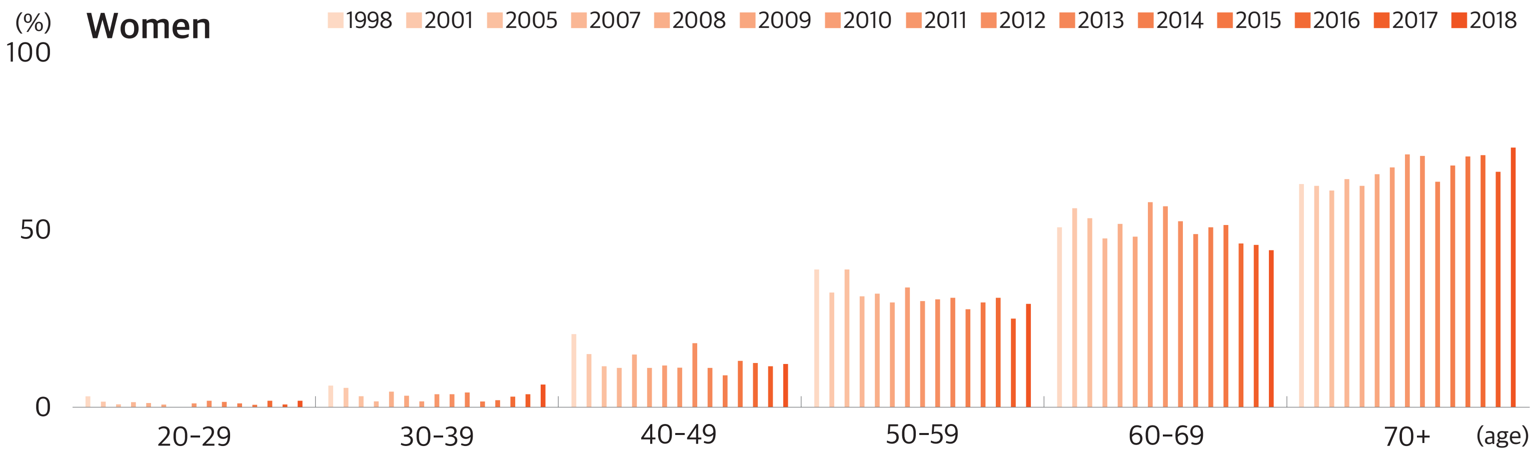

Data source: Korea National Health and Nutrition Examination Survey 1998-2018

Changes in hypertension management

Changes in awareness rate by sex and age

Changes in treatment rate by sex and age

Changes in control rate (among prevalent) by sex and age

Changes in control rate (among treated) by sex and age

Changes in awareness rate by sex and age

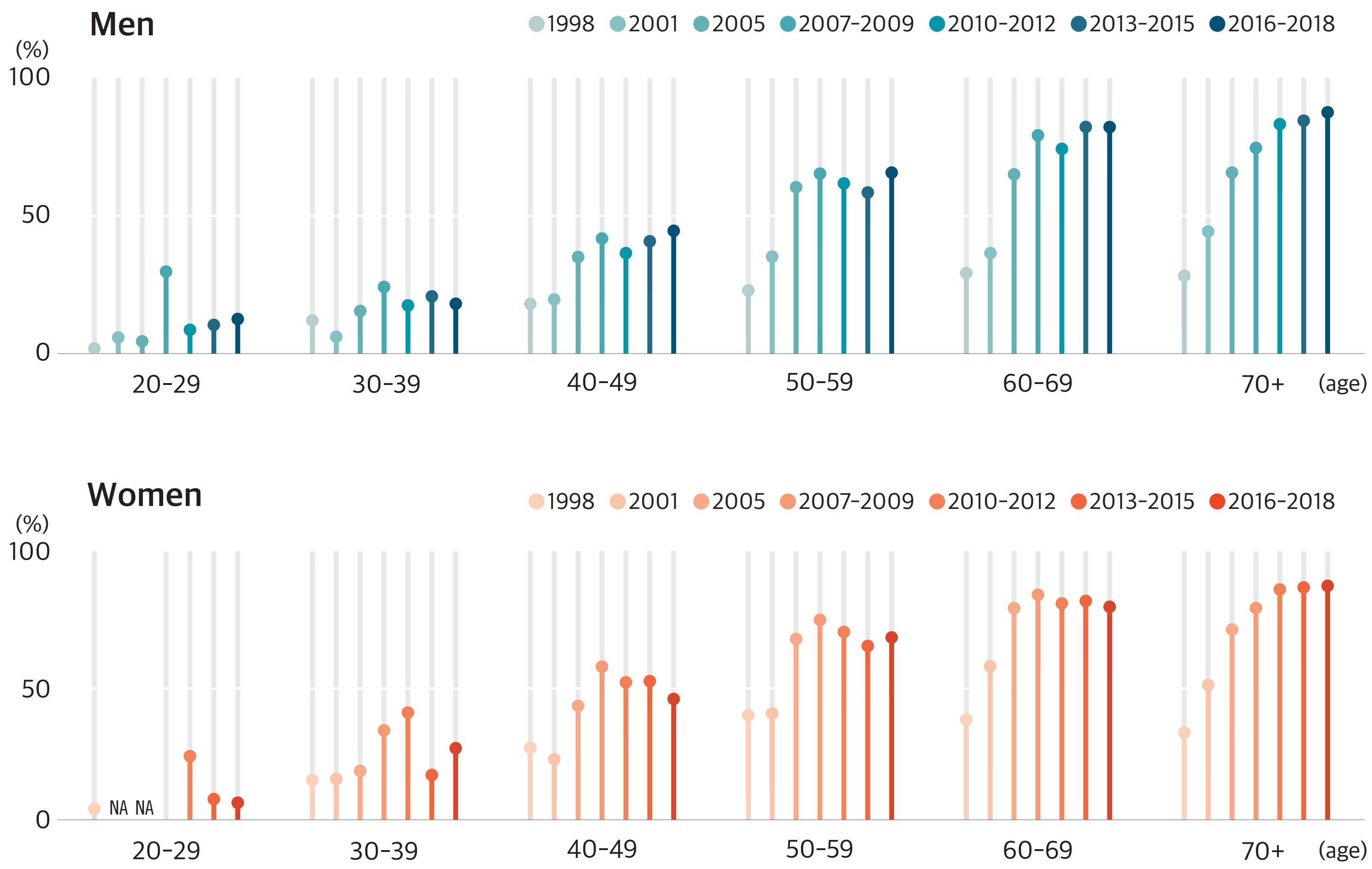

NA=Denominator less than 10

Data source: Korea National Health and Nutrition Examination Survey 1998-2018

Changes in treatment rate by sex and age

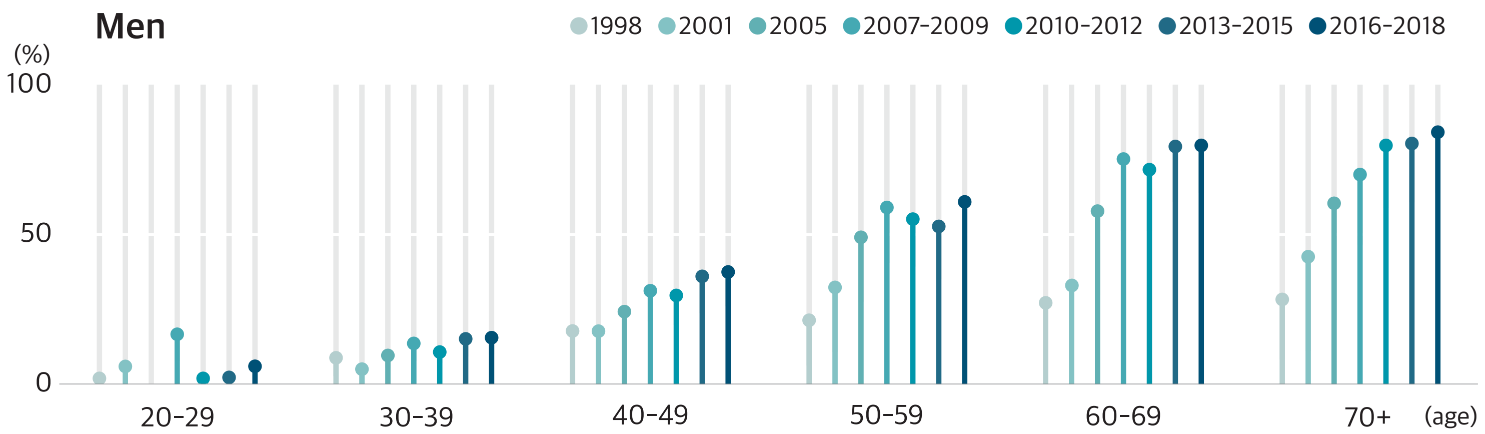

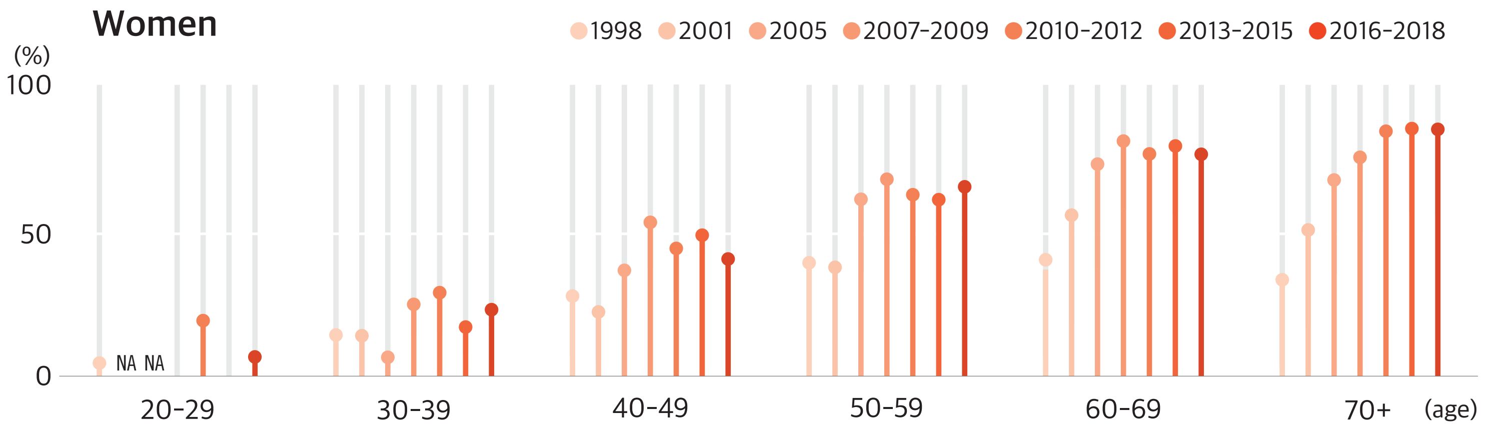

NA=Denominator less than 10

Data source: Korea National Health and Nutrition Examination Survey 1998-2018

Changes in control rate (among prevalent) by sex and age

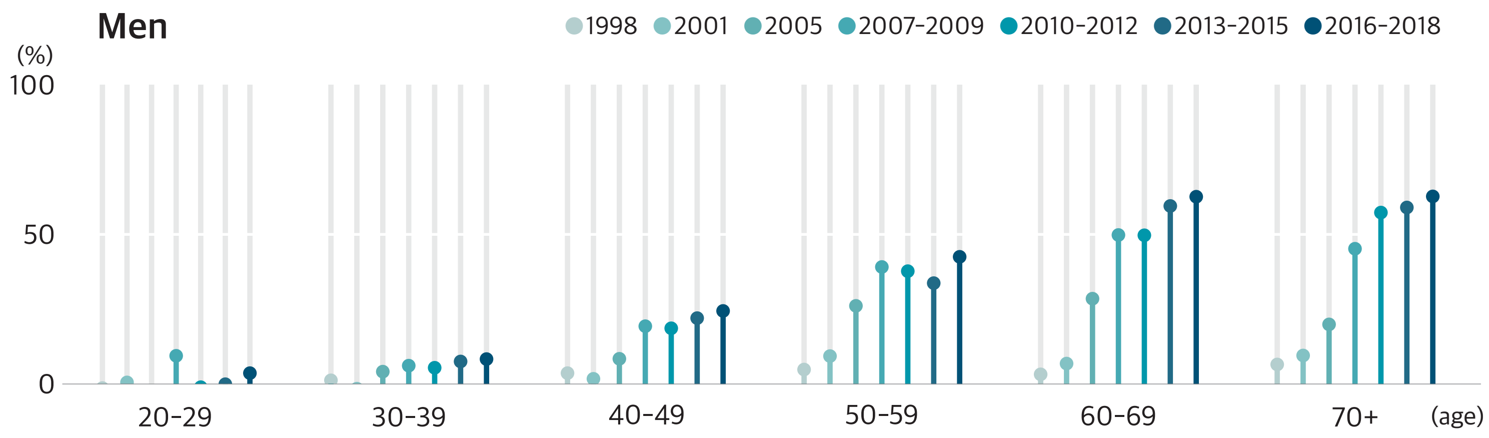

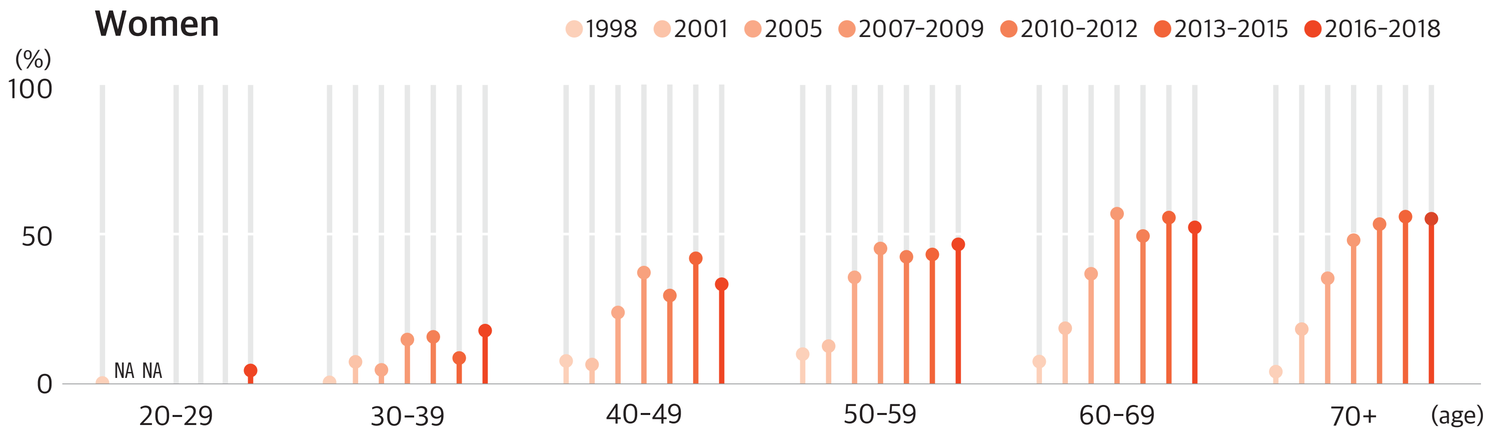

NA=Denominator less than 10

Data source: Korea National Health and Nutrition Examination Survey 1998-2018

Changes in control rate (among treated) by sex and age

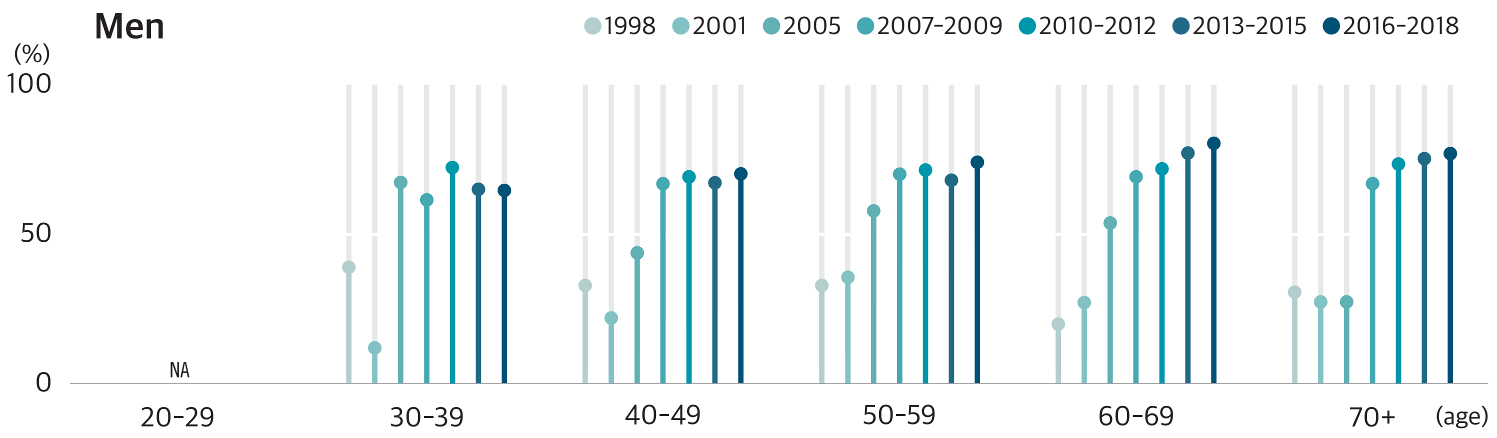

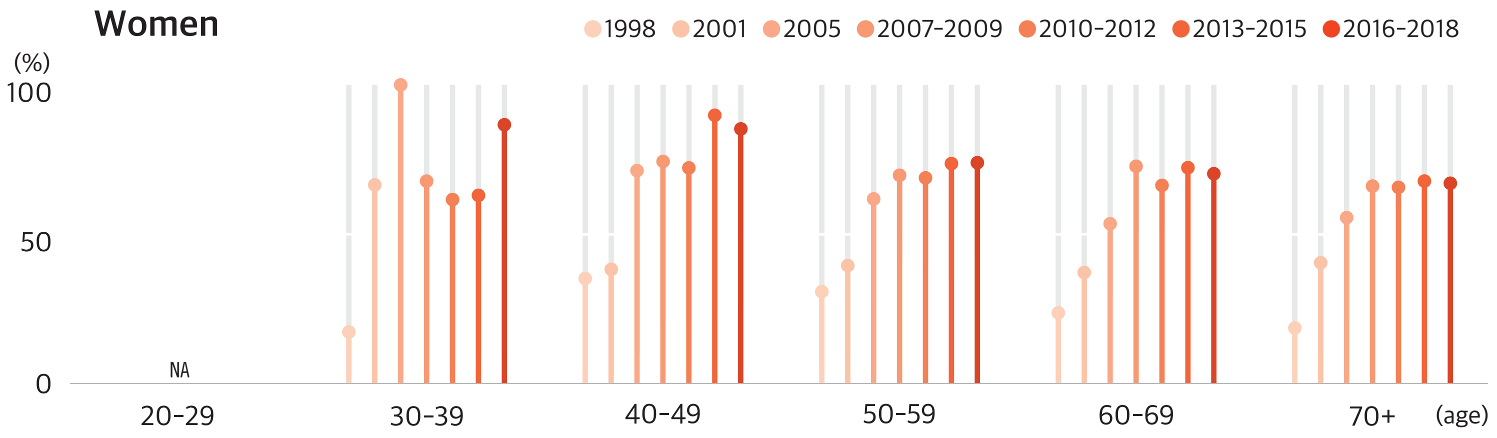

NA=Denominator less than 10

Data source: Korea National Health and Nutrition Examination Survey 1998-2018

KOREA
HYPERTENSION
FACT SHEET
2020

Healthcare utilization for hypertension

Changes in the number of healthcare users
Changes in co-treatment for dyslipidemia and diabetes
Changes in prescriptions of antihypertensive drugs
Changes in antihypertensive treatment regimen

Changes in the number of healthcare users (age 20+)

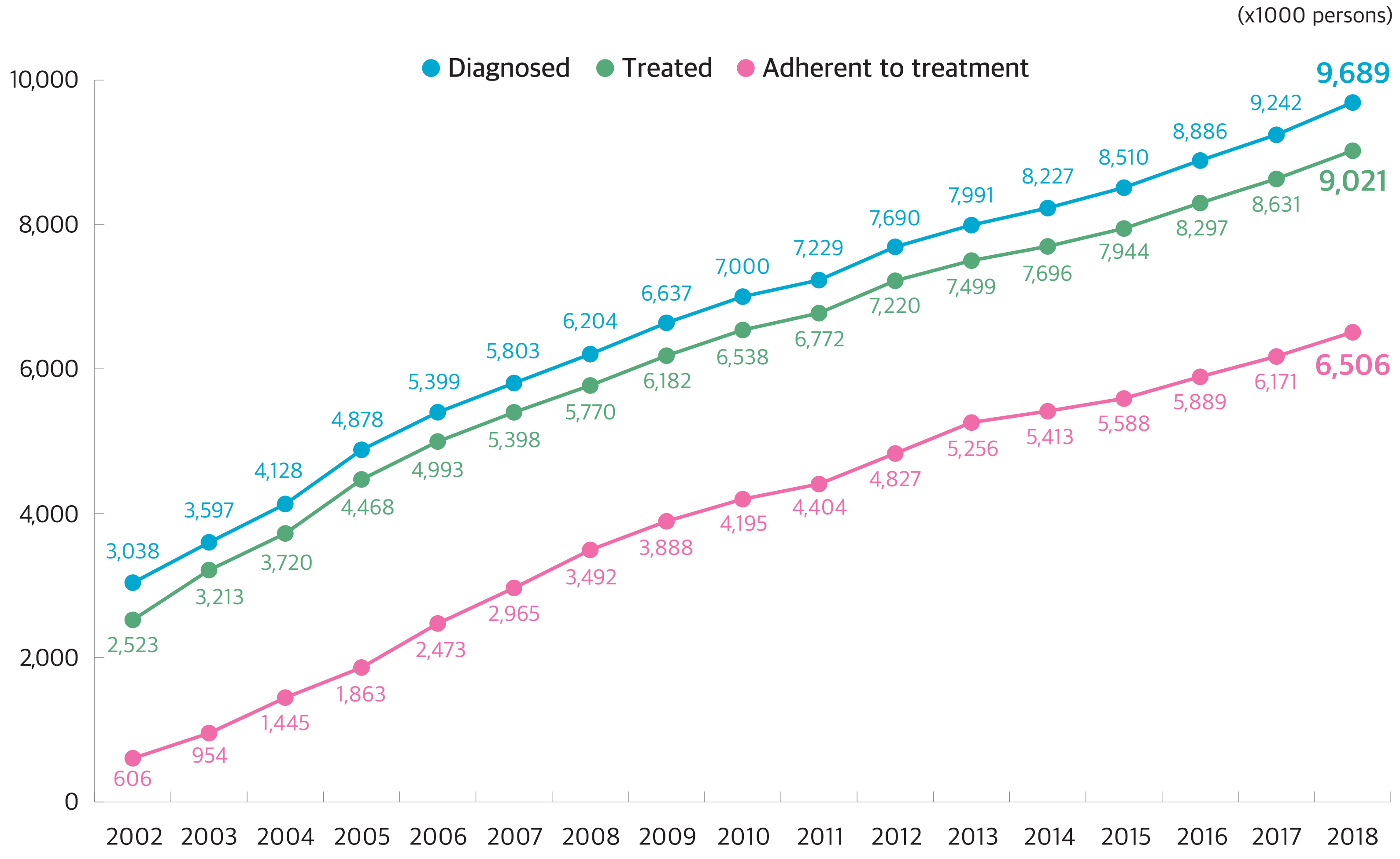

Data source: Korea National Health Insurance Big Data 2002-2018

Changes in co-treatment for dyslipidemia and diabetes (age 20+)

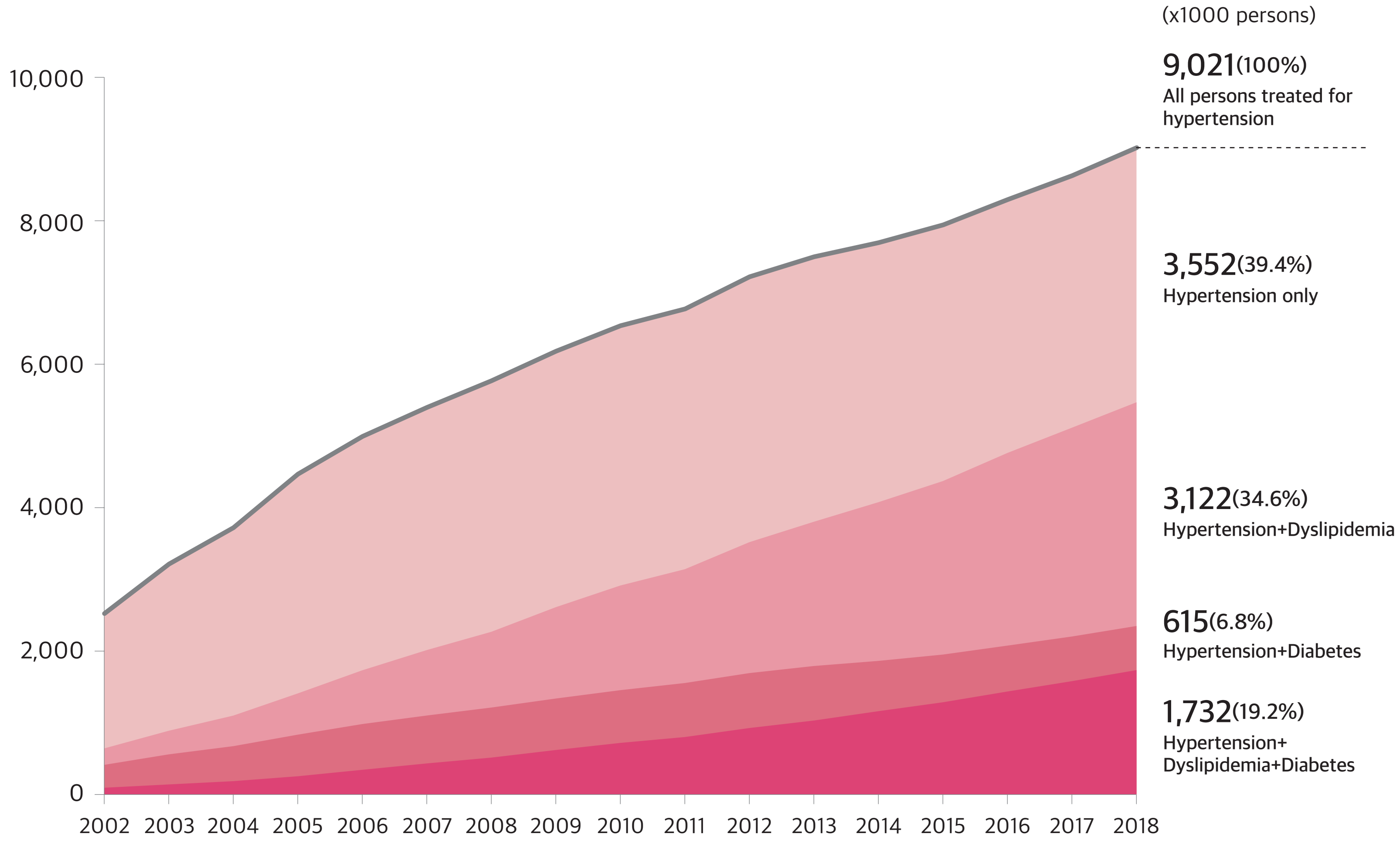

Data source: Korea National Health Insurance Big Data 2002-2018

Changes in prescriptions of antihypertensive drugs (age 20+)

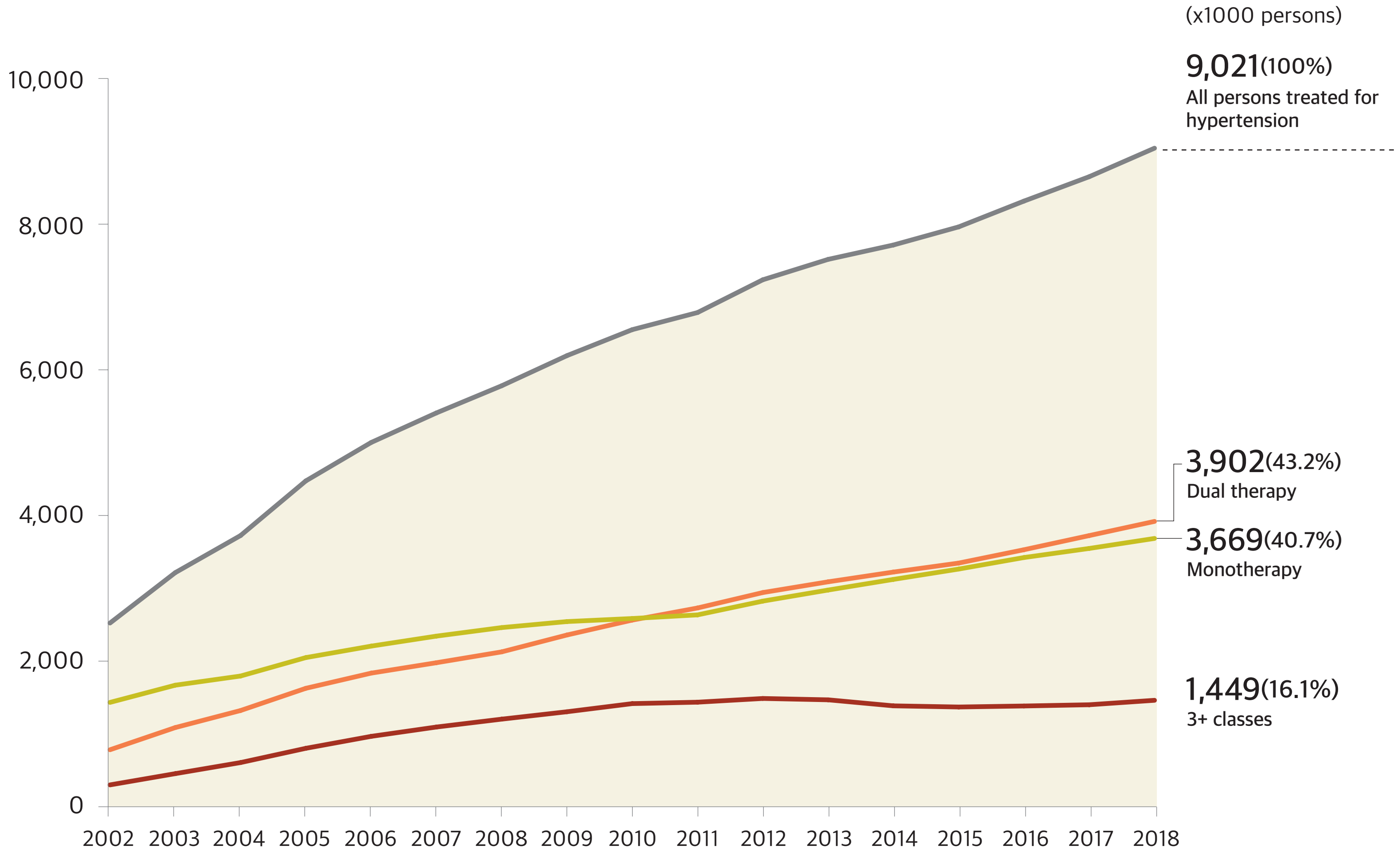

Data source: Korea National Health Insurance Big Data 2002-2018

Changes in prescriptions of antihypertensive drugs (age 20+)

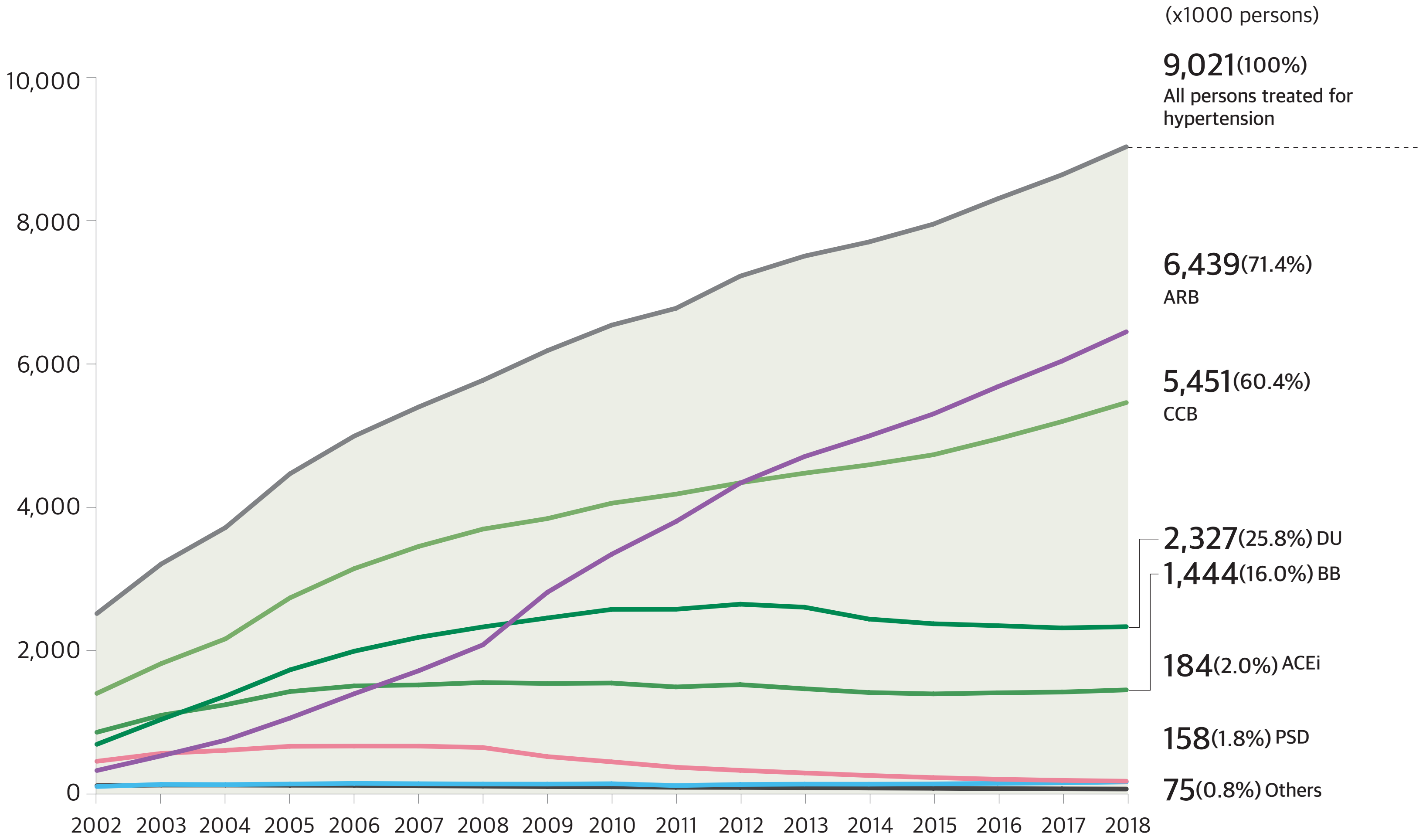

Data source: Korea National Health Insurance Big Data 2002-2018

Changes in antihypertensive treatment regimen (age 20+)

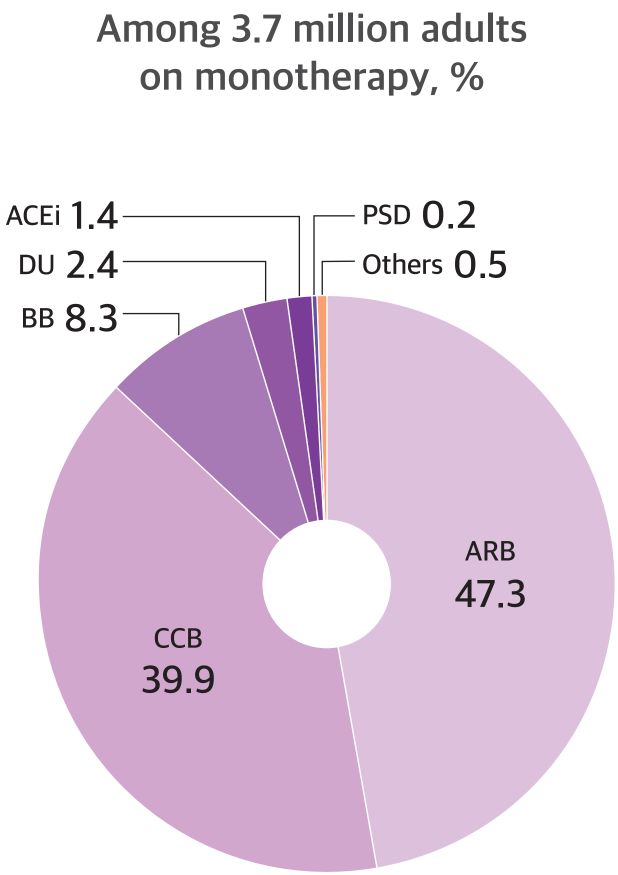

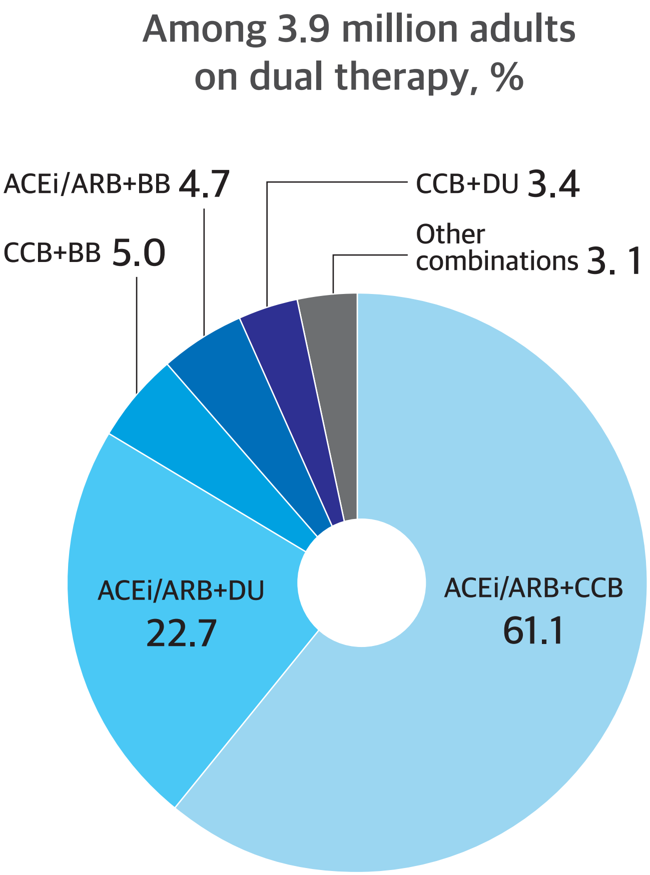

Data source: Korea National Health Insurance Big Data 2018

Changes in antihypertensive treatment regimen (age 20+)

Among 1.2 million adults on triple therapy, %

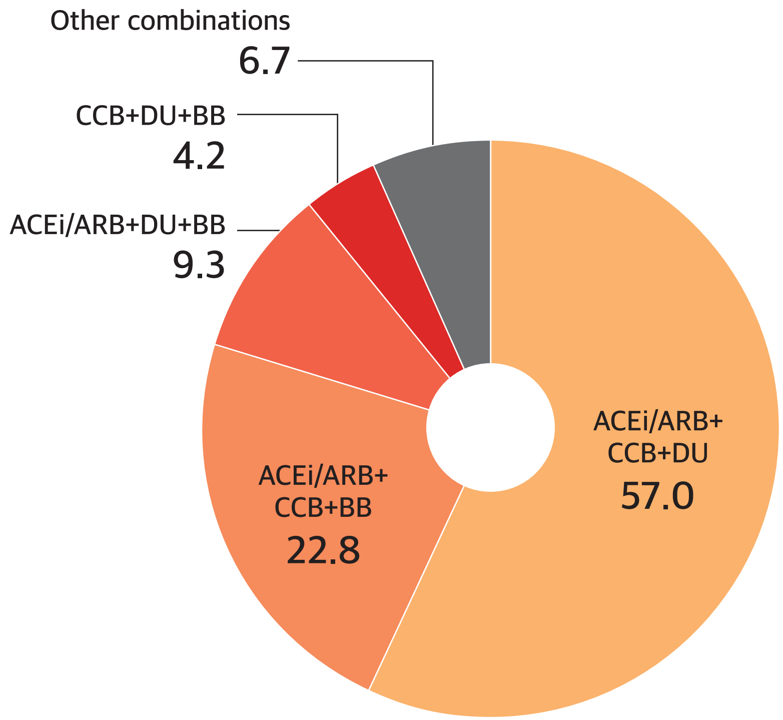

Among 0.2 million adults on 4 classes, %

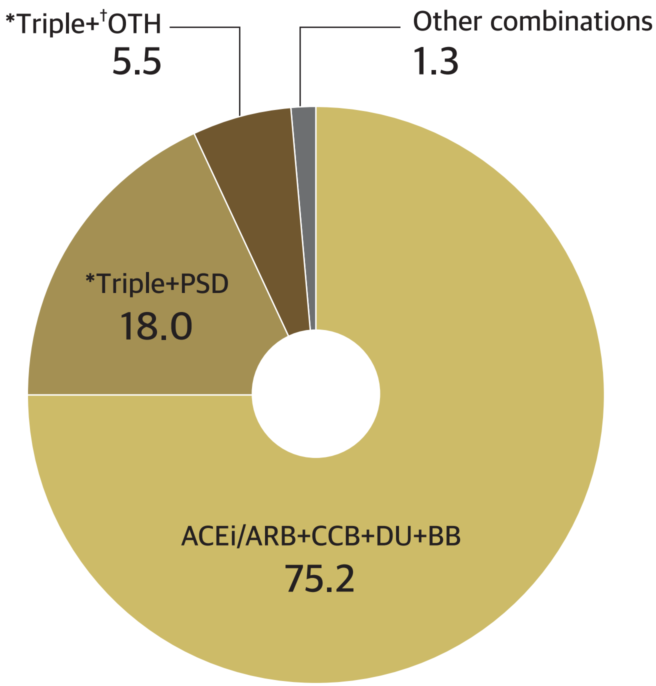

*Triple=three of the ACEi/ARB, CCB, DU, or BB
†OTH=alpha-blockers, vasodilators, etc.

Data source: Korea National Health Insurance Big Data 2018

KOREA
HYPERTENSION
FACT SHEET
2020

Management of hypertension in young adults

Summary of hypertension statistics in young adults
Changes in awareness rate in young adults
Changes in treatment rate in young adults
Changes in adherence rate (among diagnosed) in young adults
Changes in adherence rate (among treated) in young adults
Current management status in young adults

Summary of hypertension statistics in young adults (age 20-39)

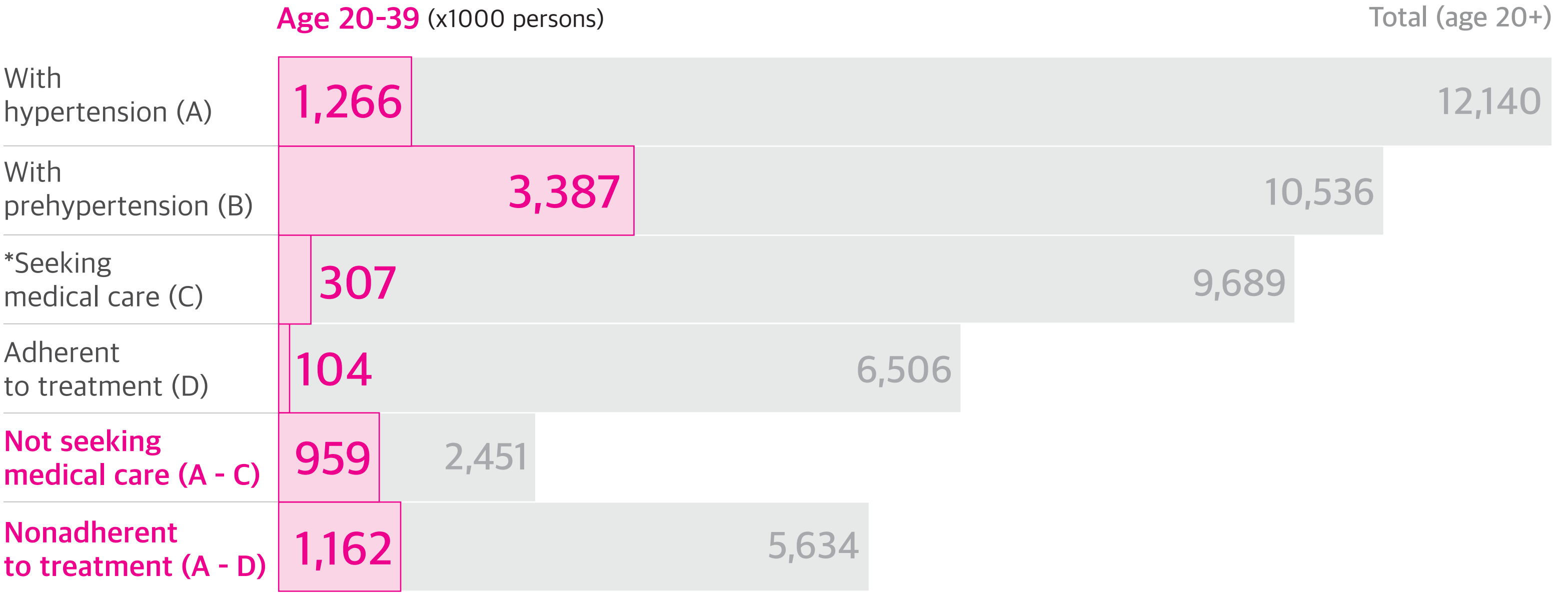

*≥1 health insurance claim for hypertension diagnosis (ICD-10: I10)

Data source: Korea National Health and Nutrition Examination Survey 2018, Korea National Health Insurance Big Data 2018

Changes in awareness rate in young adults

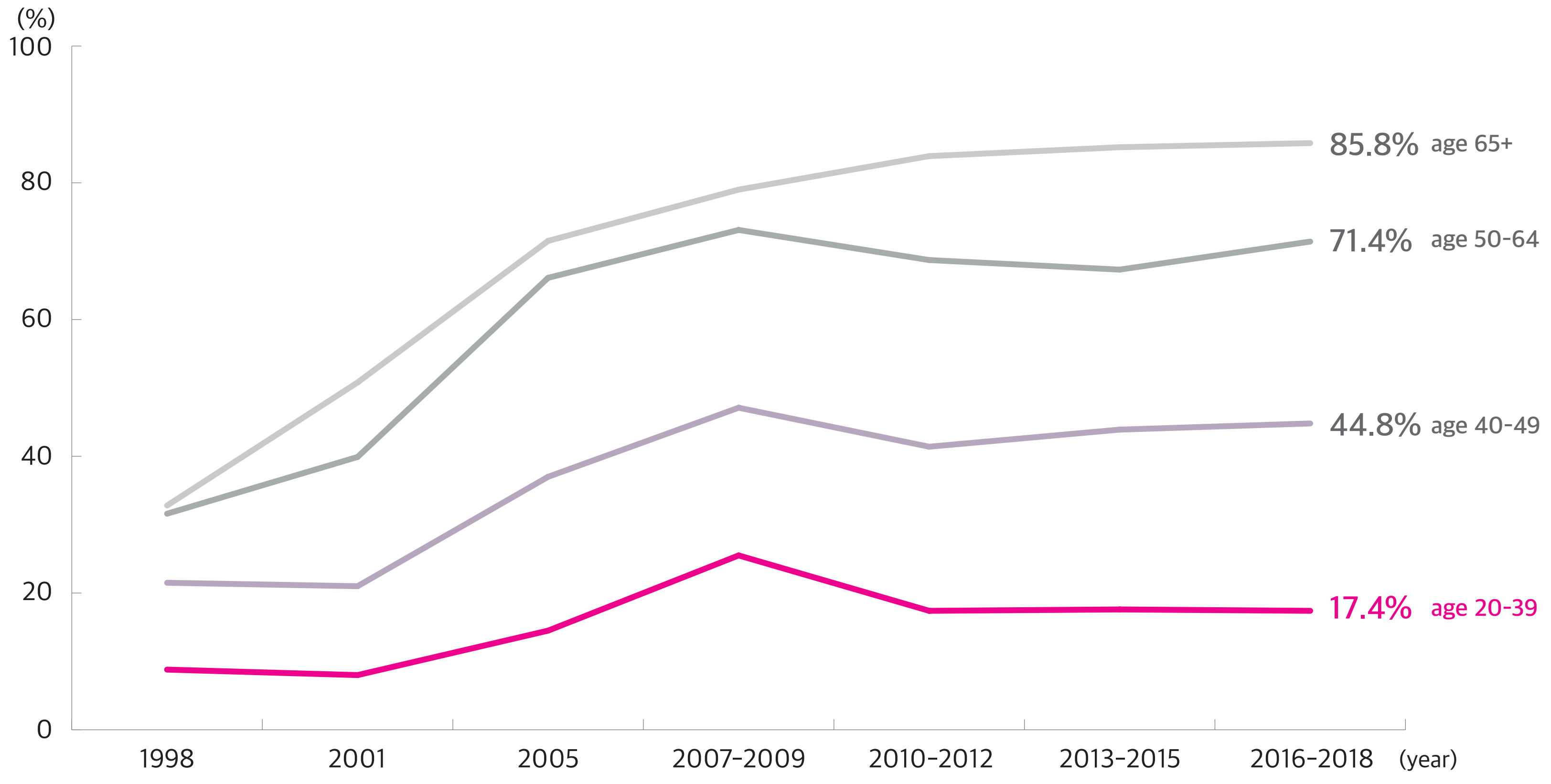

Data source: Korea National Health and Nutrition Examination Survey 1998-2018

Changes in treatment rate in young adults

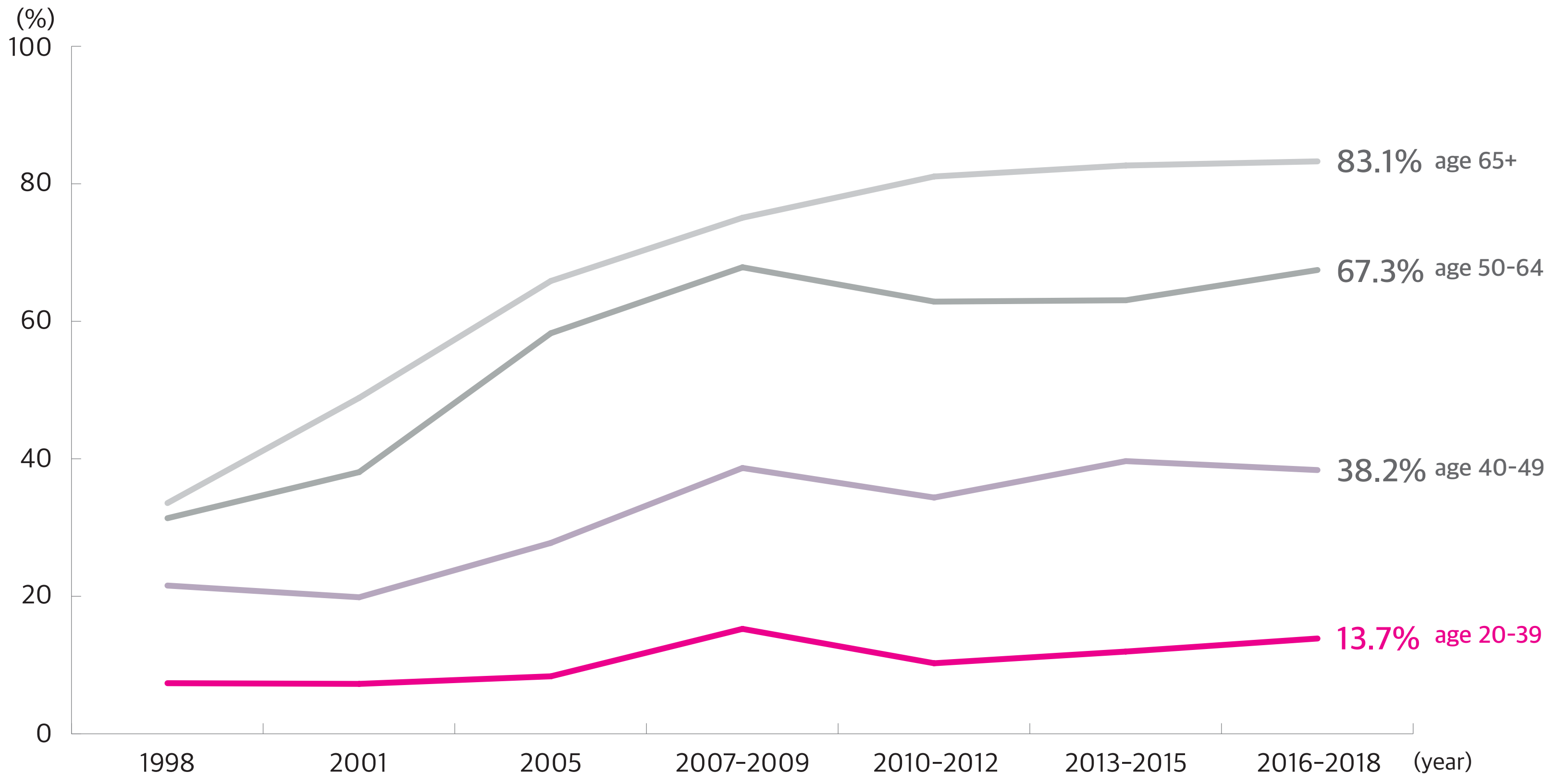

Data source: Korea National Health and Nutrition Examination Survey 1998-2018

Changes in adherence rate (among diagnosed) in young adults

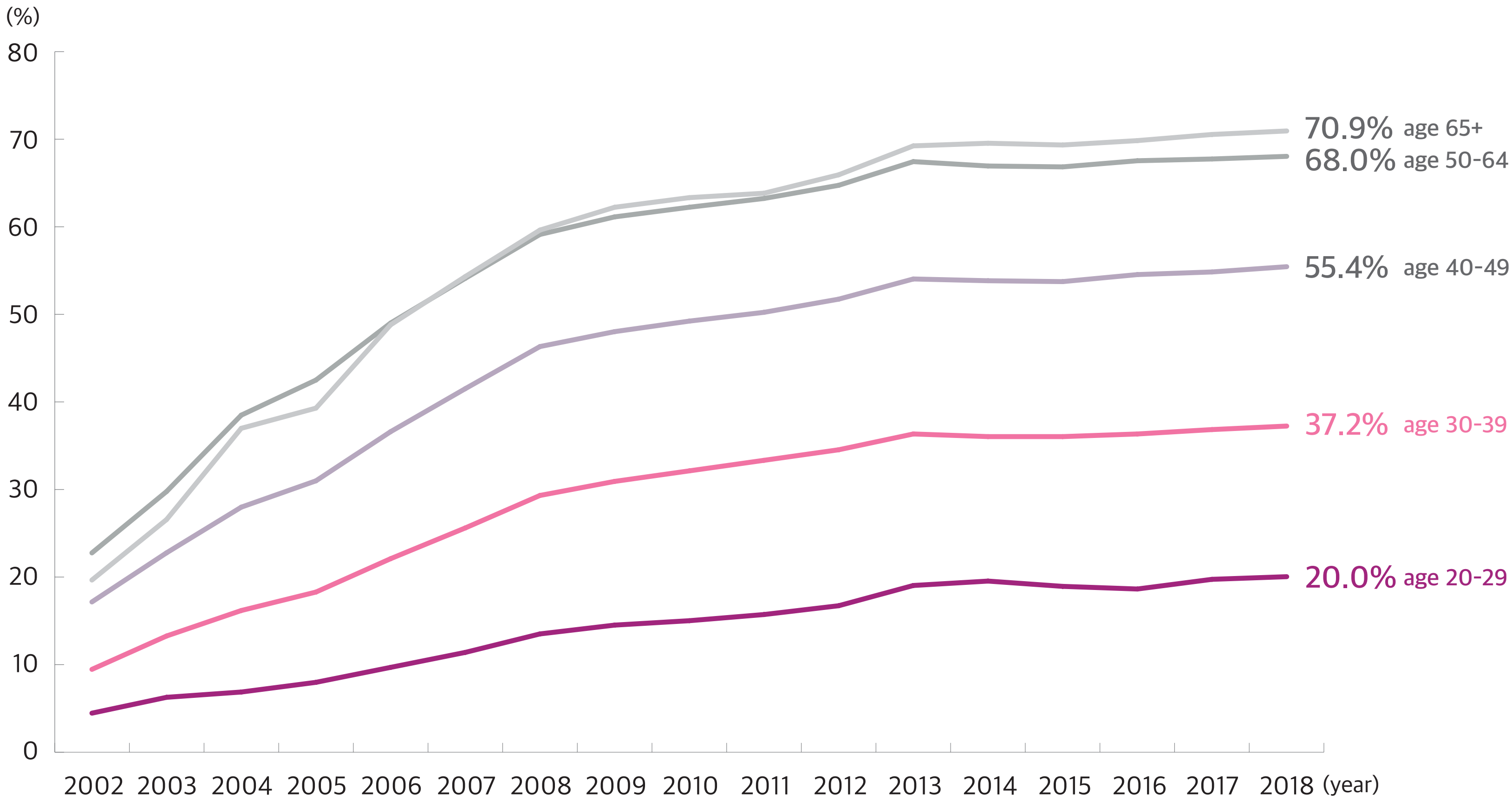

Data source: Korea National Health Insurance Big Data 2002-2018

Changes in adherence rate (among treated) in young adults

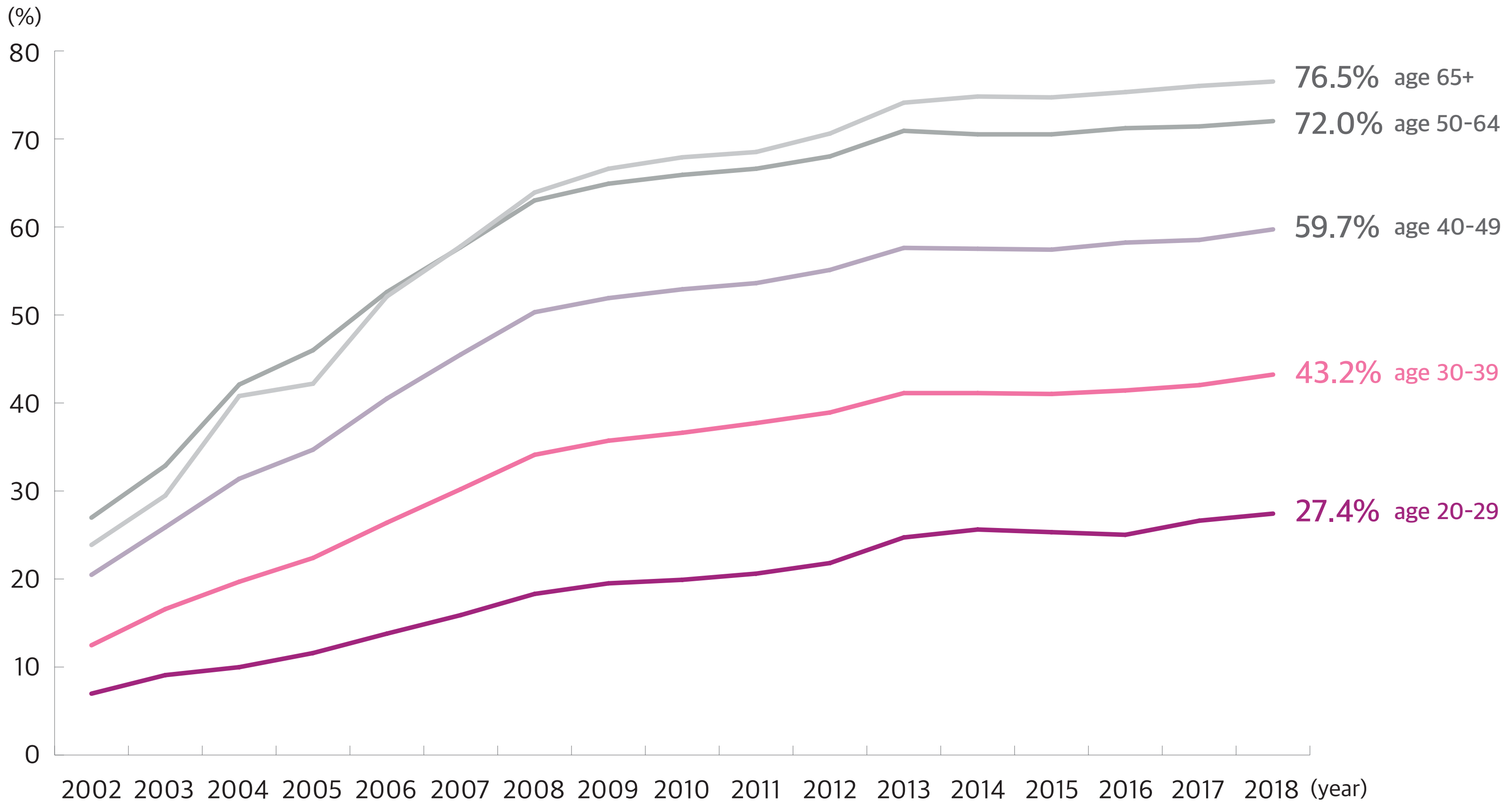

Data source: Korea National Health Insurance Big Data 2002-2018

Current management status in young adults

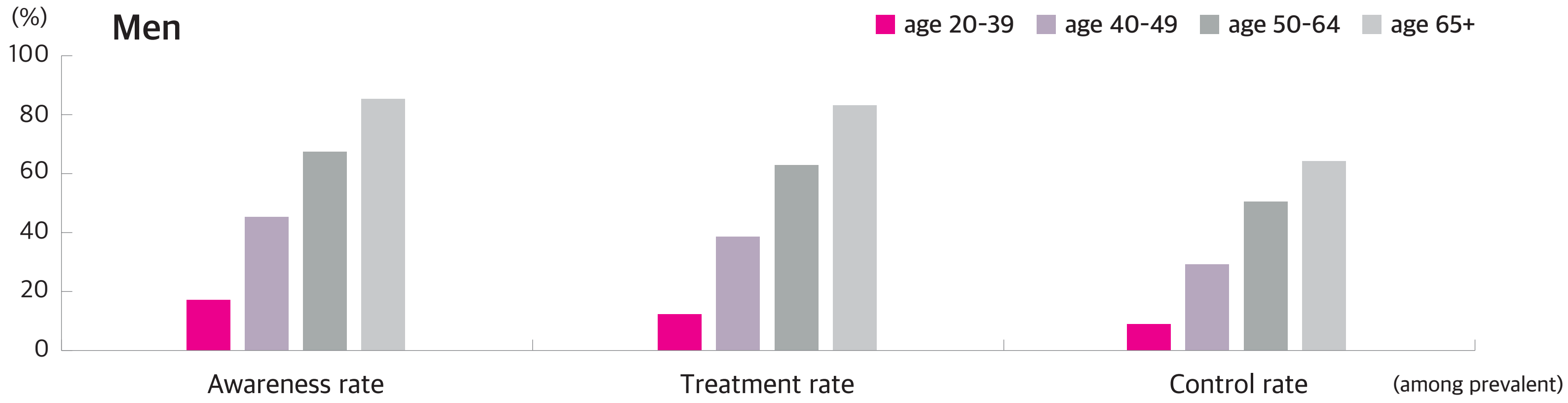

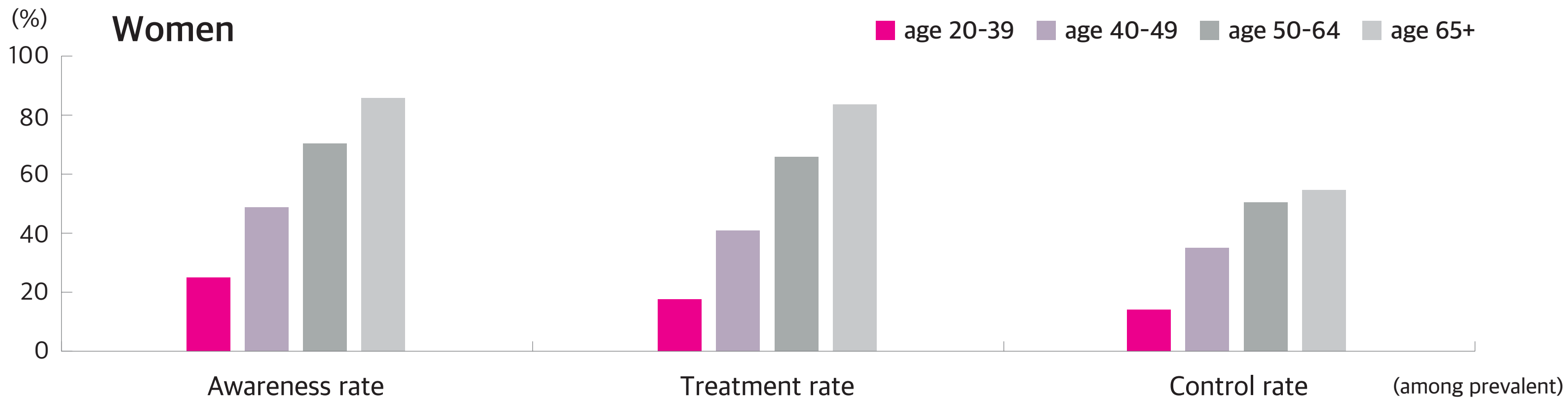

Data source: Korea National Health and Nutrition Examination Survey 2018

Current management status in young adults

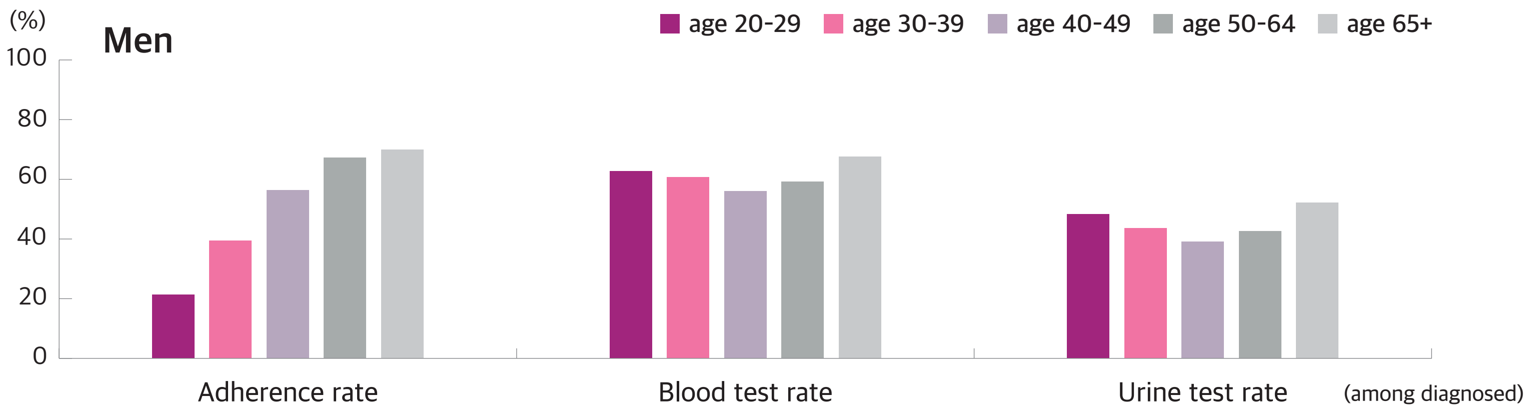

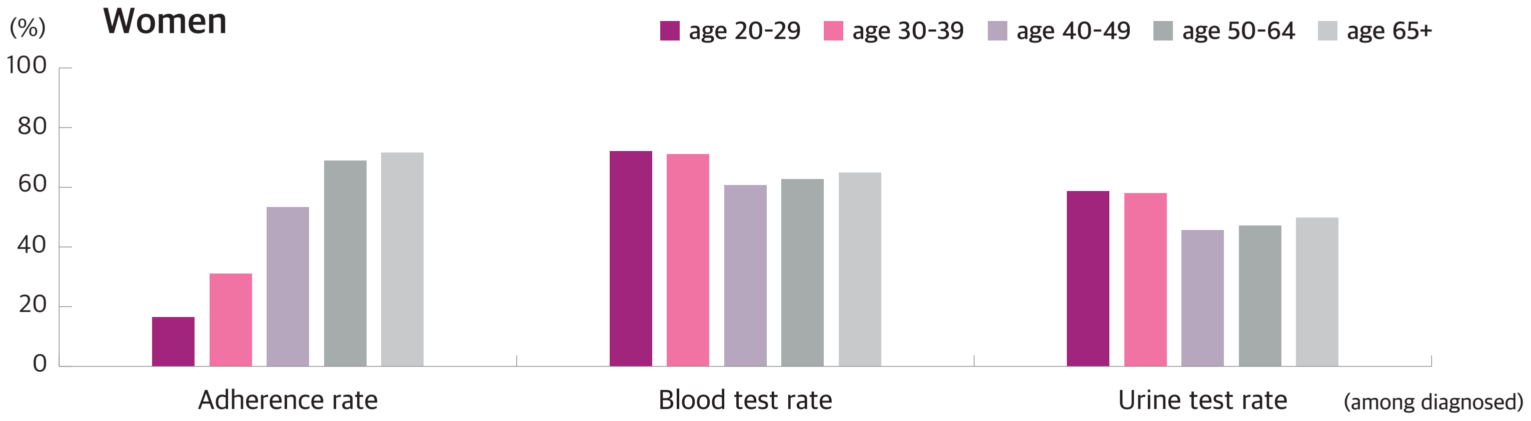

Data source: Korea National Health Insurance Big Data 2018

대한고혈압학회

THE KOREAN SOCIETY OF HYPERTENSION

This fact sheet will be published on *Clinical Hypertension*,
the official journal of The Korean Society of Hypertension

www.koreanhypertension.org
